# *Mycobacterium avium* Subsp. *paratuberculosis* and Human Endogenous Retrovirus in Italian Patients With Inflammatory Bowel Disease (IBD) and Irritable Bowel Syndrome (IBS)

**DOI:** 10.1111/imm.13923

**Published:** 2025-04-04

**Authors:** Stefano Ruberto, Alfredo Santovito, Gian P. Caviglia, Marta Noli, Davide Cossu, Davide G. Ribaldone, Demis Pitoni, Simona Frara, Elisa Tribocco, Chiara Rosso, Marta Guriglia, Ilaria Cossu, Pier A. Tovo, Massimiliano Bergallo, Leonardo A. Sechi

**Affiliations:** ^1^ Division of Microbiology and Virology, Department of Biomedical Sciences University of Sassari Sassari Italy; ^2^ Environmental Factors in Degenerative Diseases Research Group Instituto de Investigación Sanitaria del Hospital Clínico San Carlos (IdISSC) Madrid Spain; ^3^ Department of Life Sciences and Systems Biology University of Turin Torino Italy; ^4^ Department of Medical Sciences University of Turin Torino Italy; ^5^ Department of Neurology Juntendo University Tokyo Japan; ^6^ Department of Public Health and Pediatric Sciences University of Turin Turin Italy; ^7^ Pediatric Laboratory, Department of Public Health and Pediatric Sciences University of Turin, Regina Margherita Children's Hospital Turin Italy; ^8^ SC Microbiologia e Virologia Azienda Ospedaliera Universitaria Sassari Italy

**Keywords:** HERV‐K, HERV‐W, human endogenous retrovirus, IBD, IBS, MAP

## Abstract

Inflammatory bowel disease (IBD), comprising ulcerative colitis (UC) and Crohn's disease (CD) and irritable bowel syndrome (IBS) are distinct gastrointestinal disorders. *Mycobacterium avium* subspecies *paratuberculosis* (MAP) is implicated in IBD pathogenesis, while the roles of human endogenous retroviruses (HERVs) are under investigation. We aimed (a) to investigate whether the levels of humoral response to MAP‐3865c, HERV‐K envelope and HERV‐W envelope against the epitopes in IBD/IBS patients; (b) to determine the frequency of micronuclei in IBD patients and (c) to evaluate the possible correlation between genomic damage and humoral response. This study investigates antibody titres against MAP 3865c, HERV‐K env and HERV‐W env in plasma from 102 IBD, 20 IBS patients and 92 healthy controls (HCs). Micronuclei (MNi) frequency in IBD patients is assessed, correlating with humoral responses and patient genotype profiles. IBD patients exhibited elevated antibody responses to MAP 3865c, with those carrying the GA genotype for TNF‐α showing higher anti‐MAP 3865c IgG levels. A significant positive correlation was observed between MNi frequency and the humoral response against MAP 3865c in IBD patients. Higher antibody responses to HERV‐K env were detected in both IBD and IBS patients compared to HCs, with significant positive correlations found between MAP 3865c and HERV‐K env peptide responses in IBD patients. HERV‐W env antibody levels were higher in IBS patients than in HCs. Our findings highlight the association between UC and CD and immune responses targeting MAP and HERV‐Kenv. Specific genetic profiles may exacerbate inflammation, potentially amplifying genetic damage observed in IBD patients, as indicated by MNi frequencies.

## Graphical abstract

Inflammatory bowel disease (IBD), comprising ulcerative colitis (UC) and Crohn's disease (CD), is a recognised gastrointestinal disorder. *Mycobacterium avium* subspecies *paratuberculosis* (MAP) is implicated in IBD pathogenesis. Persistent exposure and active infections by MAP may contribute to the unsilencing of human endogenous retroviruses (HERV) sequences. This interaction could justify the pathogenesis of IBD by promoting HERV antigen expression and triggering autoantibody production. In addition, specific genetic profiles may exacerbate inflammation, potentially amplifying genetic damage observed in IBD patients, as indicated by MNi frequencies.

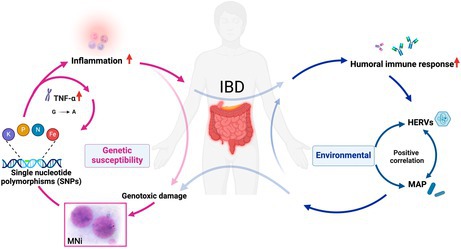

## 1 Introduction

Inflammatory bowel disease (IBD), including ulcerative colitis (UC) and Crohn's disease (CD) and irritable bowel syndrome (IBS) are two different gastrointestinal (GI) disorders [1, 2]. IBD is characterised by chronic inflammation [1], which in CD can affect the entire GI tract and in UC is limited to the inner lining of the large intestine [1]. In contrast, IBS does not cause overt inflammation [2], but often presents with symptoms similar to those of IBD, potentially related to microbial imbalances in the gut [1].

In IBD, studies have shown that dysbiosis in the gut microbiome can alter the microbial composition and contribute to microbiome disease progression [3]. Recently, low‐trace hydrogen (H_2_) detection at room temperature has emerged as a promising non‐invasive diagnostic tool for gastrointestinal disorders [4]. Elevated H_2_ levels in exhaled breath can indicate bacterial overgrowth in the gut, a feature commonly associated with IBS and could potentially provide insight into the microbial dysbiosis seen in IBD as well. Furthermore, bacterial and viral infections have been linked to the aetiology of both IBD and IBS [5, 6], either by triggering an inflammatory response in the host or by modifying the behaviour of other microbes [6]. Thus, non‐invasive methods, such as breath‐based H_2_ detection for bacterial overgrowth and antibody detection for immune responses to specific pathogens, could help monitor microbial‐related changes and provide early indicators of GI dysbiosis without requiring invasive procedures.

*Mycobacterium avium* subspecies *paratuberculosis* (MAP), primarily known as the etiological agent of Johne's disease in ruminants, has been proposed as a possible environmental factor that might contribute to the development of CD in genetically susceptible individuals [7, 8], but a direct causal relationship has not been established. The thick cell wall of the MAP bacterium is thought to enable it to withstand various chemical processes commonly used in irrigation and pasteurisation systems [8]. Published studies have shown that the presence of the MAP bacterium in at least 30% to 50% of CD patients [8, 9]. Furthermore, previous research has extensively investigated the immunopathogenic role of MAP proteins in IBD [10], showing significantly higher antibody titres against MAP‐specific proteins in individuals with CD [11, 12] and UC [13] compared to controls. Some studies have indicated that certain viruses might be associated with an increased risk of developing CD or exacerbating its symptoms in susceptible individuals [5].

Human endogenous retroviruses (HERVs) are remnants of ancient viral infections that have integrated into about 8% of the human DNA [14]. Although HERVs are not typically infectious, they can potentially influence gene expression and immune responses in various ways. In some cases, they could become activated under certain conditions, such as infection or stress, leading to the expression of viral‐like particles or proteins that might stimulate immune reactions [14]. This could explain why some HERVs are implicated in inflammatory and malignant diseases [15, 16]. Additionally, several researchers have documented a significant correlation between HERV env gene expression and various autoimmune disorders [17] and cancerous conditions [18].

The potential association between HERV and MAP, particularly in the context of GI disorders, has not been fully elucidated. Evidence suggests that MAP infection can induce DNA demethylation in specific genomic regions [19], potentially activating previously silent genes, including retroviral elements. This demethylating action could upregulate HERV expression, particularly in immune and epithelial cells, which are already susceptible to epigenetic changes in inflammatory conditions [20]. Studies indicate that demethylation of HERV LTRs can trigger increased HERV expression [21], which may play a role in the pathogenesis of GI disorders by promoting pro‐inflammatory responses or acting as superantigens that disrupt immune regulation. The observed association between HERV‐W and MAP in Type 1 diabetes [22] raises intriguing questions about whether a similar association might exist in GI disorders. This interaction might exacerbate immune responses or contribute to chronic inflammation, potentially driving the pathogenesis of conditions like IBD.

This study could provide valuable insights into microbial‐viral interactions that may drive chronic gastrointestinal inflammation, potentially advancing our understanding of the underlying mechanisms of gastrointestinal aetiology.

Furthermore, studies have explored genomic damage in patients with IBD, revealing an increase in the frequency of micronuclei (MNi) assays in this group [23]. MNi are small circular nuclei that lag during the anaphase stage of mitosis and are considered indicators of cytogenetic damage. Both IBD and IBS are influenced by genetic predisposition, inherited risks, infections and environmental conditions [3, 24]. Therefore, it could be hypothesised that cytogenetic damage, combined with alterations in environmental composition, such as bacterial infections, may contribute to different HERV expression profiles. These profiles, in turn, could influence the phenotypic manifestations of IBD and IBS.

In this study, we focused our attention on the humoral response to MAP‐3865c_125–138_, HERV‐K _env19–37_ and HERV‐W_env248–262_ in a cohort of IBS and IBD patients. We aimed to determine the presence of antibodies against these epitopes in IBD/IBS patients and investigate whether they could serve as predictors of relapse activity and correlate with an increased risk of disease progression. The second aim of the present work was to determine the frequency of MNi in IBD patients and to compare this frequency with the humoral response, considering the genotype profile of the patients.

Certain cytokine gene polymorphisms are known to be associated with inflammatory diseases and may also influence genomic stability [25, 26]. For instance, genetic variants at the interleukin IL‐10 locus have been shown to affect both innate inflammatory responses and those related to chronic diseases [27, 28]. Chronic inflammatory diseases, which are frequently associated with cellular transformation and malignancy, are often characterised by elevated systemic levels of TNF‐α, a pro‐inflammatory cytokine with broad biological effects [29, 30]. IL‐6 and TGF‐β1 are also multifunctional cytokines that play critical roles in cell proliferation, differentiation, and inflammatory pathways, as well as in acute‐phase responses [31, 32].

Our hypothesis is that, in patients with IBD, these specific cytokine gene polymorphisms could play dual roles in modulating both the immune response and the extent of DNA damage. These polymorphisms were chosen due to their established roles in inflammatory pathways and potential impact on genomic stability, which aligns with our study's objective to investigate the genetic and inflammatory factors contributing to IBD pathology. We hope that this added context provides clarity on our selection criteria.

## 2 Materials and Methods

### 2.1 Patients

A total of 102 IBD patients, 20 IBS patients, and 92 age‐ and gender‐matched healthy controls (HCs) were recruited from the “A.O.U. Città della Salute e della Scienza” hospital, in Turin, Italy. The inclusion criteria for IBD patients involved an age over 18 years and a definite diagnosis of Crohn's disease (CD) or ulcerative colitis (UC) according to ECCO criteria [33]. The IBD group consisted of 52 patients with CD and 50 patients with UC. Concerning IBS population, aged 18–65 years, was diagnosed as subtype D according to Rome IV criteria [34]. The demographic characteristics of the participants are summarised in Table 1.

**TABLE 1:** Demographic characteristics of patients and healthy controls.

		No.	Sex (no. of men/women)	Age (year) median ± SD
HC		92	49 / 43	44 ± 13.6
IBD	UC	50	35 / 15	53 ± 14.0
	CD	52	29 / 23	42 ± 15.1
IBS		20	7 / 13	36 ± 13.3

Abbreviations: CD, Crohn's disease; HC, healthy controls; IBD, inflammatory bowel disease; IBS, Irritable bowel syndrome; UC, ulcerative colitis.

### 2.2 Blood Sample Collection

Blood samples were collected from the participants by venipuncture into tubes containing EDTA. The plasma samples were isolated by standard Ficoll Histopaque (Sigma‐Aldrich, St. Louis, MO, USA) gradient centrifugation and subsequently stored at −20°C.

### 2.3 Peptides

Synthetic peptides MAP‐3865c_125–138_ (MIAVALAGL) from the transmembrane protein of MAP (UniProt accession no. Q73T54), HERV‐K_19–37_ (VWVPGPTDDRCPAKPEEEG) from the envelope protein of HML‐2 (UniProt accession no. O42043), and HERV‐W_248–262_ (NSQCIRWVTPPTQIV) from the envelope protein (UniProt accession no. Q9UQF0) were synthesised with a purity greater than 90% and purchased from LifeTein (South Plainfield, NJ, USA). All peptides were reconstituted at 10 mM in dimethylsulphoxide.

### 2.4 Enzyme‐Linked Immunosorbent Assay (ELISA)

Plasma antibodies recognising retroviral peptides were detected using an indirect enzyme‐linked immunosorbent assay (ELISA) for immunoglobulin G (IgG) detection, following the protocol of a previously published study [35]. The experiment was conducted with an equimolar concentration of 10 μM for each peptide. Both negative and positive control plasma were included to validate the protocol. The negative control consisted of plasma from healthy individuals or from individuals known not to have antibodies to the retroviruses of interest, ensuring that the assay specifically detects the targeted antibodies without interference from non‐specific binding. The positive control consisted of plasma from individuals with known antibody positivity to the retroviral peptides, confirming that the assay could reliably detect specific IgG responses. Additionally, assays were run in duplicate to confirm the reproducibility of the results, and both inter‐assay and intra‐assay variability were assessed to ensure consistent performance across different experiments. Each sample was analysed in triplicate, and its reactivity was evaluated as the mean optical density at 405 nm.

### 2.5 Lymphocyte Culture

The MNi assay involved n. 29 IBD subjects (mean age ± SD, 46 ± 14.5, n. 16 males and n. 13 females). For each subject, 0.3 mL of venous blood was cultured in 25 cm^2^ flasks containing 6 mL of RPMI‐1640 medium supplemented with 20% foetal calf serum (FCS), 2% of the mitogenic agent PHA, l‐glutamine (2 mM), and antibiotics (100 IU/mL penicillin, and 100 μg/mL streptomycin). The cultures were incubated for 72 h at 37°C in a humidified atmosphere with 5% CO_2_. To block cytokinesis, cytochalasin‐B was added to the cultures at a concentration of 6 μg/mL after 44 h of incubation. After 72 h of incubation at 37°C, the cells were collected by centrifugation and treated for 10 min with a pre‐warmed mild hypotonic solution (75 mM KCl). Following centrifugation and removal of the supernatant, the cells were fixed with a fresh mixture of methanol/acetic acid (3:1 v/v). The treatment with the fixative was repeated three times. Finally, the supernatant was discarded, and the pellet was dissolved in a minimal volume of fixative and seeded on the slides. MNi were detected by conventional staining with 5% Giemsa (pH 6.8) prepared in Sörensen buffer.

### 2.6 Cytokinesis‐Block Micronucleus (MNi) Assay

Genomic damage was measured using the MNi assay, a fast and inexpensive test that can detect both clastogenic and aneugenic properties of a single chemical or a mixture of compounds. MNi are whole chromosomes or chromosome fragments that fail to migrate to anaphase during mitosis, thus resulting in visible extranuclear bodies in interphase nuclei. The MNi assay also consents to evaluate the frequency of nuclear buds (NBUDs), which represent the elimination of amplified DNA or excess chromosomes from aneuploid cells [36].

The frequency MNi and the number of NBUDs were measured in 1000 binucleated lymphocytes with well‐preserved cytoplasm per subject. Additionally, a total of 1000 lymphocytes per donor were scored to determine the cytokinesis‐block proliferation index (CBPI), according to the following formula: 1 × *N*1 + [2 × *N*2 + 3 × (*N*3 + *N*4)]/*N*, where *N*1–*N*4 represent the number of cells with 1–4 nuclei, respectively, and *N* is the total number of cells evaluated.

### 2.7 DNA Extraction and Genotyping

Heparinized vacutainers were used to collect 5–10 mL of peripheral blood from each subject. Then, the vacutainers were stored at −20°C prior to analysis. DNA extraction was conducted by using a salting‐out procedure: 0.5 mL of peripheral blood was added to red cell lysis buffer (10 mM Tris pH 7.6; 5 mM MgCl_2_ and 10 mM NaCl), gently shaken for 30 s and centrifuged at 14000 rpm for 1 min. The pellet was re‐suspended in a solution consisting of white blood cells (10 mM Tris pH 7.6 [Merck, Milan, Italy]; 10 mM EDTA [Merck, Milan, Italy] and 50 mM NaCl [Merck, Milan, Italy]), 10 μL of SDS 10% (Merck, Milan, Italy) and 30 μL of proteinase K (Merck, Milan, Italy). After incubation at 55°C for 1 h, 200 μL of saturated sodium acetate was added to the solution. The samples were vigorously shaken and centrifuged at 14000 rpm for 5 min. Subsequently, 0.5 mL of isopropanol for DNA precipitation was added to the supernatant solution and, after centrifugation at 14000 rpm for 1 min, 0.5 mL of 70% ethanol was added to remove salt from the pellet. After 30–60 min at room temperature, the pellet was resuspended in 50 μL of ultrapure distilled water. All subjects were genotyped for *TNF‐α* (G/A −308), *IL10* (G/A −1082, C/T −819), *TGF‐β* (C/T Codon 10, G/C Codon 25) and *IL6* (G/C −174) polymorphisms, using primers and methodologies as described in Table 2. PCR reactions were performed in a 25 μL volume containing about 10 ng DNA (template), with a final concentration of 1× Reaction Buffer (Thermo Fisher Scientific Inc., Waltham, MA, USA), 1.5 mM of MgCl_2_ (Thermo Fisher Scientific Inc., Waltham, MA, USA), 5% of DMSO (Thermo Fisher Scientific Inc., Waltham, MA, USA), 250 μM of dNTPs (Thermo Fisher Scientific Inc., Waltham, MA, USA), 0.5 μM of each primer and 1 U/sample of Taq DNA polymerase (Thermo Fisher Scientific Inc., Waltham, MA, USA). Cycles were set as follows: 35 cycles, 1 min at 95°C, 1 min at 60°C, 1 min at 72°C and a final extension step of 10 min at 72°C. Amplification products were detected by ethidium bromide (Merck, Milan, Italy) staining after 3% agarose gel (Società Italiana Chimici, Rome, Italy) electrophoresis.

**TABLE 2:** Primers and annealing temperatures for gene polymorphisms analysed in the present study.

Gene	Main function protein	Reference SNP	Sequence	PCR product (bp)	Reference
IL‐6 (−174, G>C) – Antisense primer – G‐sense primer – C‐sense primer	Pro‐inflammatory	rs1800796	5′‐TCGTGCATGACTTCAGCTTTA‐3′ 5′‐AATGTGACGTCCTTTAGCATG‐3′ 5′‐AATGTGACGTCCTTTAGCATC‐3′	190	Zakharyan et al. [37]
IL 10 −1082 (G>A) – Antisense primer – G‐sense primer – A‐sense primer	Anti‐inflammatory	rs1800896	5′‐AGTGCCAACTGAGAATTTGG‐3′ 5′‐CTACTAAGGCTTCTTTGGGAG‐3′ 5′‐ACTACTAAGGCTTCTTTGGGAA‐3′	258	Perrey et al. [38]
TNF‐α (−308, G>A) – Antisense primer – G‐sense primer – A‐sense primer	Pro‐inflammatory	rs1800629	5′‐TCTCGGTTTCTTCTCCATCG‐3′ 5′‐ATAGGTTTTGAGGGGCATGG‐3′ 5′‐AATAGGTTTTGAGGGGCATGA‐3′	184	Perrey et al. [38]
TGF‐β_1_ (Codon 10, T>C) – Antisense primer – C‐sense primer – T‐sense primer	Tissue repair, anti‐inflammatory	rs1800471	5′‐TCCGTGGGATACTGAGACAC‐3′ 5′‐GCAGCGGTAGCAGCAGCG‐3′ 5′‐AGCAGCGGTAGCAGCAGCA‐3′	241	Perrey et al. [38]

*Note*: For all genes, the annealing temperature was 60°C and the used methodology was ARMS‐PCR.

### 2.8 Statistical Analysis

Data analysis was conducted using GraphPad Prism 8.4.1 software (GraphPad Software, San Diego, CA, USA). The Shapiro–Wilk test was performed to assess the normality of the data distribution. Non‐parametric Mann–Whitney and Fisher's exact tests were employed for comparison between the IBD, IBS, and HC groups. Receiver operating characteristic (ROC) curves were generated to evaluate the accuracy of the experiments. The cut‐off for positivity in each ELISA was established at 95% specificity, and the corresponding sensitivity was calculated accordingly. Spearman's correlation test was applied to assess the correlation between the antibody response of the analysed peptides and the numbers of MNs. Pearson's *χ* ^2^ test was conducted for Hardy–Weinberg equilibrium and to compare allele or genotype frequencies. A *p*‐value ≤ 0.05 was considered statistically significant.

## 3 Results

### 3.1 Antibody Responses to MAP and Genetic Associations in IBD and IBS Patients

**FIGURE 1:**
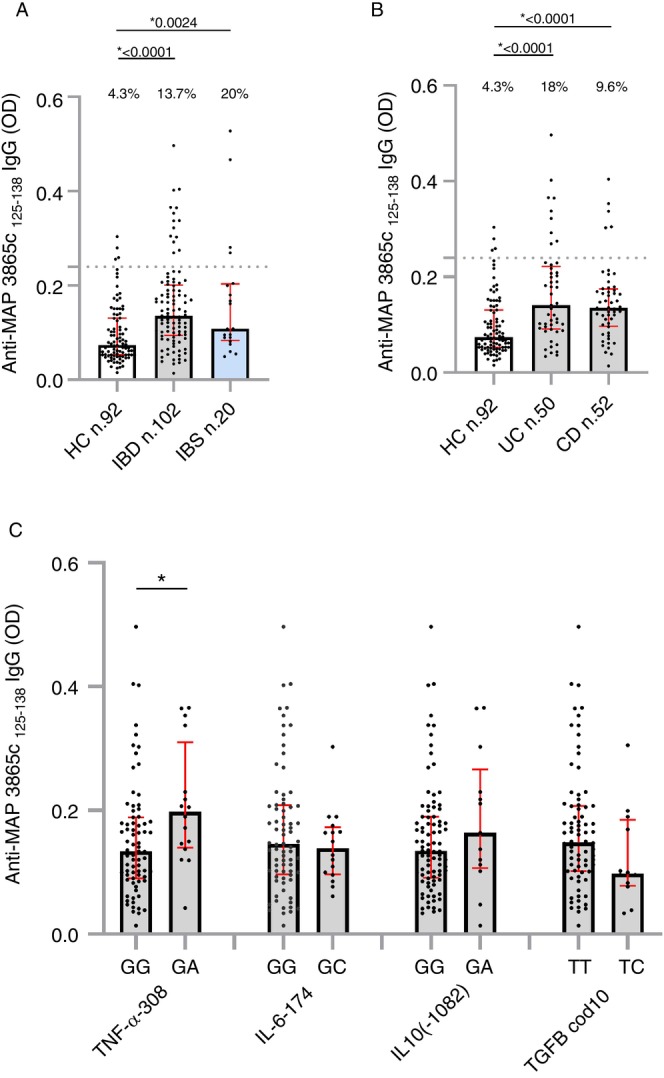
Antibody titres against MAP 3865c_125–138_ and genotype correlation in patients with gastrointestinal disorders and healthy controls. Graphs displaying the cutoff values (dashed lines) and the median with interquartile range for plasma levels of anti‐MAP 3865c_125–138_ antibodies in (A) inflammatory bowel disease (IBD; grey), irritable bowel syndrome (IBS; blue), and healthy controls (HC; white) and (B) ulcerative colitis (UC) and Crohn's disease (CD). Each dot represents the titre of a patient. The percentage of positive for each group is shown at the top of each graph. (C) Antibody titres against MAP 3865c_125–133_ detected in IBD patients according to the genotype of the studied polymorphism. Statistical analyses were performed using Shapiro–Wilk, Mann–Whitney and Fisher's exact tests. *p* ≤ 0.05 was considered statistically significant.

The antibody responses to MAP‐3865c_125–138_ were significantly higher in IBD patients (median: 0.13, interquartile range [IQR]: 0.20–0.09) and in IBS patients (median: 0.11, IQR: 0.20–0.08), compared to HCs (median: 0.07, IQR: 0.13–0.04, *p* < 0.01) (Figure 1A). Similarly, the anti‐MAP‐3865c_125–138_ IgG response reported significantly higher levels in UC (median: 0.14, IQR: 0.22–0.09) and in CD patients (median: 0.13, IQR: 0.17–0.09) than HCs (*p* < 0.0001) (Figure 1B).

Table 3 summarises the alleles and genotypes frequencies of the different cytokine genes that were analysed. All gene polymorphisms were in the Hardy–Weinberg equilibrium. Additionally, IBD patients with the “GA” genotype for TNF‐α showed significantly higher levels of anti‐MAP‐3865c IgG response than the individuals with the “GG” genotype (*p* = 0.01). No significant difference in the humoral response against MAP‐3865c_125–138_ peptide was found according to the genotype for the other studied polymorphisms (Figure 1C).

**TABLE 3:** Allele and genotype frequencies of the studied inflammatory gene polymorphisms in the IBD population. (*n* = 93).

Gene polymorphisms	Allele	*N*	Frequency	Genotype	*N*	Frequency	HWE *p*
TNF‐α–308	G	167	0.9	GG	75	0.81	0.99
	A	19	0.01	GA	17	0.18	
				AA	1	0.01	
IL‐6–174	G	170	0.91	GG	77	0.83	0.66
	C	16	0.09	GC	16	0.17	
				CC	0	0.00	
IL10 (–1082)	G	173	0.93	GG	80	0.86	0.76
	A	13	0.07	GA	13	0.14	
				AA	0	0.00	
TGFB cod10	T	174	0.93	TT	81	0.87	0.8
	C	12	0.7	TC	12	0.13	
				CC	0	0.00	

### 3.2 Correlations Between Antibody Response and Micronucleus Assay

**FIGURE 2:**
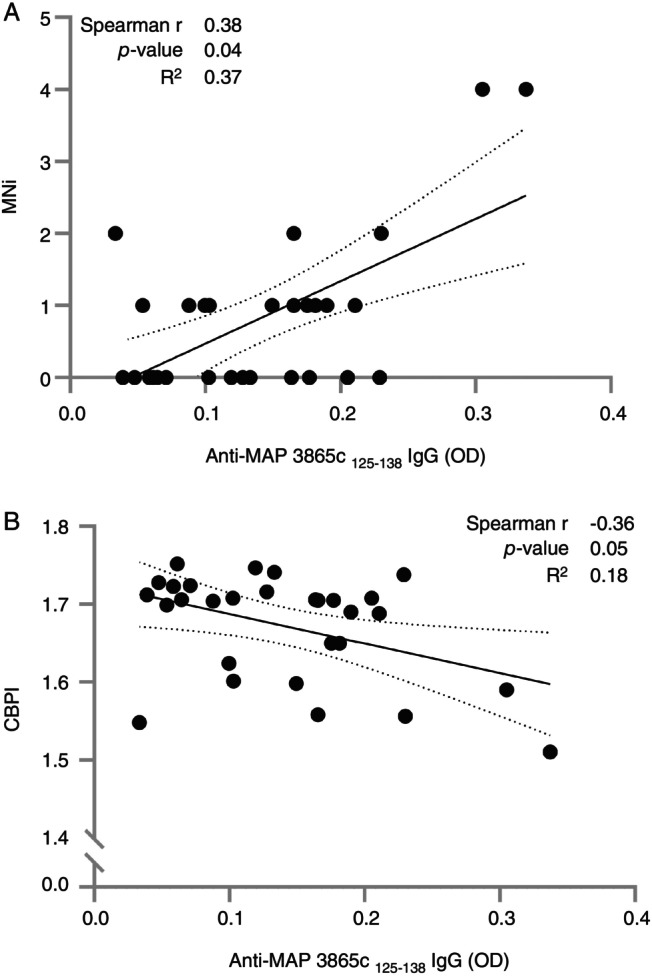
Correlations between MAP 3865c_125–138_ antibody titres, micronuclei and proliferation index in IBD patients. Scatter plot of the correlations between MAP 3865c_125–138_ antibody titres and (A) micronuclei (MNi) and (B) cytokinesis‐block proliferation index (CBPI) in IBD patients. Spearman's correlation and simple linear regression (*R* ^2^) were used for analysis. Statistical significance was set at *p* ≤ 0.05.

According to the micronucleus assay, a significant positive correlation was found between the MNi frequency and the humoral response levels against MAP‐3865c_125–138_ peptide (Figure 2A) in IBD patients. In line with this result, a significant negative correlation was found between the cytokinesis‐block proliferation index and the antibody responses to MAP 3865c_125–138_ peptide (Figure 2B).

### 3.3 Antibody Responses to HERV‐K and HERV‐W Envelope Peptides in IBD and IBS Patients

**FIGURE 3:**
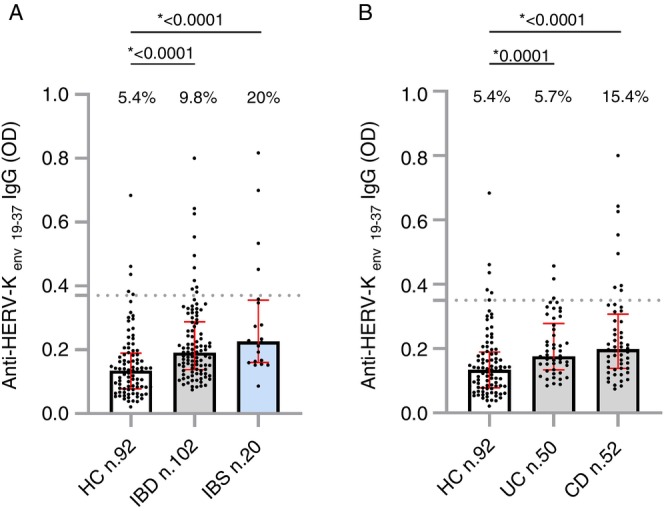
Antibody titres against HERV‐K_env19–37_ in patients with gastrointestinal disorders and healthy controls. The graphs displaying the cutoff values (dashed lines) and the median with interquartile range for plasma levels of anti‐HERV‐K_env19–37_ antibodies in (A) inflammatory bowel disease (IBD; grey) irritable bowel syndrome (IBS; blue) and healthy controls (HC; white) and (B) ulcerative colitis (UC) and Crohn's disease. The percentage of positive for each group is shown at the top of each graph. Statistical analyses were performed using Shapiro–Wilk and Mann–Whitney tests. *p* ≤ 0.05 was considered statistically significant.

The antibody responses to HERV‐K_env19–37_ were significantly higher in IBD patients (median: 0.18, IQR: 0.28–0.13) and in IBS patients (median: 0.22, IQR: 0.35–0.16), compared to HCs (median: 0.12, IQR: 0.19–0.07, *p* < 0.0001) (Figure 3A). Similarly, the anti‐HERV‐K_env19–37_ IgG response reported significantly higher levels in UC (median: 0.17, IQR: 0.27–0.13) and in CD patients (median: 0.18, IQR: 0.30–0.13), than HCs (*p* < 0.0001) (Figure 3B). No significant difference in the humoral response against HERV‐K_env19–37_ peptide was found according to the genotype for all studied polymorphisms (data not shown).

**FIGURE 4:**
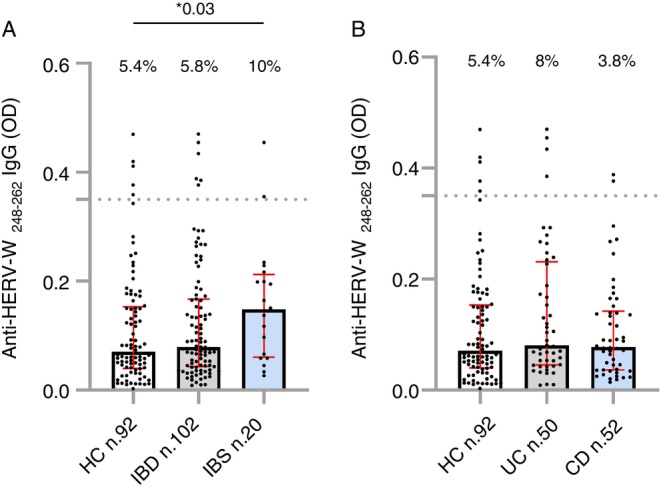
Antibody titres against HERV‐W_env248–262_ in patients with gastrointestinal disorders and healthy controls. Graphs displaying the cutoff values (dashed lines), the median with interquartile range for plasma levels of anti‐HERV‐W_env248–262_ antibodies in (A) inflammatory bowel disease (IBD; grey), irritable bowel syndrome (IBS; blue) and healthy controls (HC; white) and (B) ulcerative colitis (UC) and Crohn's disease (CD). Each dot represents the titre of a patient. The percentage of positive individuals for each group is displayed above each graph. Statistical significance was determined using Shapiro–Wilk and Mann–Whitney tests, with *p* ≤ 0.05 considered statistically significant.

The antibody responses to HERV‐W_env248–262_ were significantly higher in IBS patients (median: 0.14, IQR: 0.21–0.05) than in HCs (median: 0.07, IQR: 0.15–0.04) (Figure 4A). However, no significant differences in the humoral response against HERV‐W_env248–262_ were found in IBD patients (median: 0.8, IQR: 0.16–0.04) in both UC (median: 0.08, IQR: 0.23–0.04) and the CD group (median: 0.07, IQR: 0.14–0.03), compared to HCs (Figure 4B). No significant difference in the humoral response against the HERV‐W_env248–262_ peptide was found according to the genotype for all studied polymorphisms (data not shown). The frequency distribution of positive and negative cases in populations with IBD and IBS according to the IgG response for all epitopes studied is finally shown in Table 4.

**TABLE 4:** Distribution of positive and negative cases in the IBD versus IBS population according to the anti‐MAP‐3865c_125–138_, anti‐HERV‐K_env19–37_ and anti‐HERV‐W_env248–262_ IgG response.

−/+ (%)	MAP‐3865c	HERV‐K_env19–37_	HERV‐W_env 248–262_
IBD	88/14 (13.7)	92/10 (9.8)	96/6 (5.8)
IBS	14/4 (20)	16/4 (20)	18/2 (10)

### 3.4 Correlations between MAP and HERV‐K antibody responses in IBD patients and HCs

**FIGURE 5:**
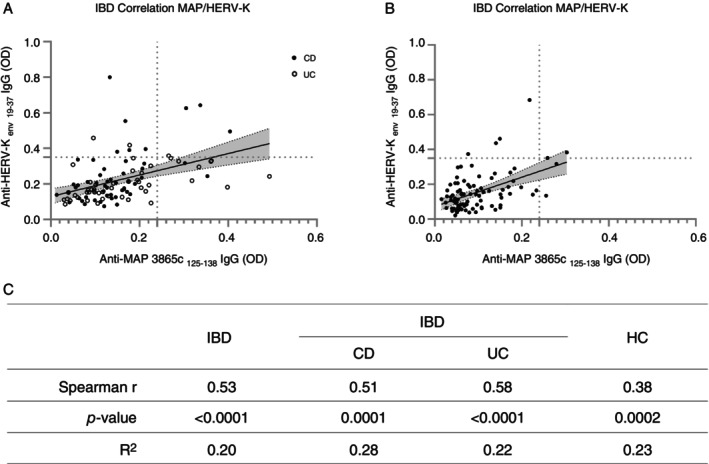
Correlations between MAP 3865c_125–138_ and HERV‐K_env19–37_ antibody titres in patients with gastrointestinal disorders and healthy controls. Scatter plot showing correlations between MAP 3865c_125–138_ and HERV‐K_env19–37_ antibody titres in (A) IBD patients, ulcerative colitis (UC), Crohn's disease (CD), and (B) healthy controls (HCs). (C) Spearman's correlation and simple linear regression (*R* ^2^) were used for analysis. Statistical analysis was set at *p* ≤ 0.05.

Significant positive correlations were found between the humoral response levels against MAP 3865c_125–138_ and HERV‐K_env19–37_ peptides, in IBD patients (Figure 5A) and HCs (Figure 5B) according to Spearman's correlation and simple linear regression (Figure 5C).

## 4 Discussion

In this study, we investigated MAP‐3865c, HERV‐K_env_, and HERV‐W_env_ serology in IBD and IBS patients. Our findings revealed significantly increased levels of antibody titre against MAP‐3865c_125–138_ and HERV‐K_env19–37_ in both IBD (UC and CD) and IBS patients, with specifically increased antibody levels against HERV‐W_env248–262_ in IBS patients. Meta‐analyses conducted on IBD patients corroborate these results, showing a higher prevalence of MAP‐specific antibodies compared to controls [39, 40, 41]. Notably, illness severity does not correlate with MAP antibody levels, and evidence suggests that surgery or anti‐MAP treatments do not affect the disease progression [40].

There are many potential mechanisms of the intestinal disease associated with the MAP, but sufficient evidence is lacking to carry out further studies [7]. Animal model studies have shown that the MAP can increase the permeability of intestinal cells by loosening the junctions between them. It also recruits macrophages to the site where an infection is taking place [42]. It is believed that the MAP can activate various inflammatory pathways in the intestine, leading to the development of granulomatous inflammation [43]. The degree to which the inflammatory response is triggered by the MAP can be influenced by a genetic component. This is why only a small number of people infected with the MAP develop CD [7].

*De facto*, we found that individuals carrying the TNF −308A SNP exhibit significantly higher anti‐MAP IgG titres compared to those with the GG genotype. TNF, a pro‐inflammatory cytokine, is influenced by the −308 (rs1800629) SNP, where the minor allele (A) enhances TNF‐α production [44], potentially amplifying the anti‐MAP immune response. This SNP has implications in predicting non‐responsiveness to anti‐TNF therapy in CD patients [45] and has been theorised to possibly increase susceptibility to MAP infection and reactivation of latent tuberculosis among anti‐TNF treated individuals [46].

The inflammatory process associated with IBD has been implicated in the induction of DNA damage, loss of cellular membrane integrity, and cytogenetic alterations [47, 48]. The increase in cytogenetic damage observed via the MNi assay moderately correlates with an increased IgG anti‐MAP response in IBD patients, suggesting a link between inflammation severity and genomic instability, potentially relevant to cancer development [48]. Consistent with our findings, Castro‐Garza reported increased genomic instability, evaluated by the MNi assay, in macrophages during *mycobacterial* infection [49]. Additionally, a reduction in the cytokinesis‐block proliferation index negatively correlated with an elevated IgG anti‐MAP response in IBD patients, indicating decreased cellular viability alongside increased inflammatory activity.

In IBD and IBS, various infectious agents have been proposed as potential triggers [3], although the conclusive evidence remains incomplete. Our study revealed significantly elevated antibody titres against HERV‐K in both IBD and IBS patients, aligning with previous findings of altered HERV expression patterns in inflamed gut tissues compared to healthy individuals [5, 50]. Specifically, HERV‐K env's involvement in autoimmune diseases and carcinogenesis is well documented [51].

The results agree with Tovo et al. (2024), who in a study of a cohort of Italian IBD patients showed increased HERV‐K gene expression in CD and UC compared with HCs, with no differences between UC and CD patients [52]. Conversely, higher anti‐HERV‐W antibody levels were observed only in IBS subjects, although this result could be potentially influenced by the small size of the IBS sample. This finding parallels reports of HERV‐W env down‐regulation during acute CD colon inflammation [50].

Several studies evidenced the role of environmental factors, cytokines, and co‐infections like *Human herpesvirus* (HHV) and *Epstein–Barr virus* (EBV) in the activation of HERV genes, possibly through epigenetic modifications [53].

Studies revealed that EBV infections can increase the risk of developing lymphoproliferative conditions in IBD patients after using immunosuppressive drugs. The presence of EBV in the colonic *mucosa* could contribute to the high clinical activity in IBD patients. Therefore, positivity to EBV infection in IBD patients could contribute to a reactivation of HERV sequences [51]. The exact mechanism in human viral infection of HERV remains unclear [20], it is difficult to ascertain whether HERVs are accelerators or a bystander in the aetiology of IBD.

Persistent exposure and active infections by MAP may contribute to the unsilencing of HERV sequences. This interaction could justify the pathogenesis of IBD by promoting HERV antigen expression and triggering autoantibody production. Additionally, MAP has been linked to HERV associations in autoimmune diabetes [54] and multiple sclerosis (MS) [55], supporting our hypothesis of MAP‐induced HERV activation in IBD.

Furthermore, pro‐inflammatory cytokines such as TNF‐α may upregulate HERV‐K expression through interactions involving interferon regulatory factor 1 (IRF1) and interferon‐stimulated response elements (ISREs) with HERV‐K long terminal repeats (LTRs) [21]. Thus, increased MAP‐induced inflammation might enhance HERV‐K expression, further stimulating the anti‐HERV‐K response in IBD patients. This positive feedback loop exacerbates inflammation and genomic damage [56], as indicated by the strong correlation between MNi and the anti‐MAP IgG response in IBD.

Limitations of our study include the relatively small sample size of IBS patients and challenges in elucidating definitive genotypic differences due to low allele frequency detection. Expanding our sample size across both IBS and IBD cohorts would strengthen the reliability of our findings regarding cytokine gene allele variations and inflammatory conditions.

In addition, since gene polymorphisms are associated with inflammatory conditions, considering the geographical distribution of patients could clarify the susceptibility of the population to autoimmune inflammatory diseases.

In conclusion, our study reinforces the connection between inflammatory diseases like UC and CD and the immune response directed against MAP and HERV‐K_env_. Therefore, the MAP exposure and activation of HERVs could influence the clinical evolution of the disease, allowing their potential application as IBD prognostic factors.

Specific genetic profiles may intensify inflammation and contribute to the genetic damage observed in IBD patients, as evidenced by MNi. Increasing the sample size and investigating HERV envelope expression could provide further insights into MAP's potential role in reactivating dormant viral sequences.

## Author Contributions

S.R. drafted the manuscript. S.R., A.S., I.C. and M.N. performed the experiment, data curation, formal analysis, methodology, software. G.P.C., D.G.R. and D.P. recruited the samples, reviewing and editing. C.R., M.G., S.F., E.T., D.C., P.A.T., M.B. and L.A.S. critically revised the draft. All authors read and approved the final manuscript.

## Ethics Statement

The study received ethical approval from the “Comitato Etico Interaziendale A.O.U. Città della Salute e della Scienza di Torino‐A.O. Ordine Mauriziano‐A.S.L. Città di Torino” (approval code n. 0056924) and was conducted in accordance with the principles outlined in the World Medical Association's Helsinki declaration.

## Consent

Informed consent was received from every participant.

## Conflicts of Interest

The authors declare no conflicts of interest.

## Data Availability

The data that support the findings of this study are available from the corresponding author upon reasonable request.
